# Development of a PBPK model to quantitatively understand absorption and disposition mechanism and support future clinical trials for PB‐201

**DOI:** 10.1002/psp4.12964

**Published:** 2023-04-20

**Authors:** Miao Zhang, Zihan Lei, Ziheng Yu, Xueting Yao, Haiyan Li, Min Xu, Dongyang Liu

**Affiliations:** ^1^ Drug Clinical Trial Center Peking University Third Hospital Beijing China; ^2^ Department of Pharmaceutical Sciences, School of Pharmacy and Pharmaceutical Sciences University at Buffalo, The State University of New York Buffalo New York USA; ^3^ Department of Obstetrics and Gynecology Peking University Third Hospital Beijing China; ^4^ Department of Cardiology and Institute of Vascular Medicine Peking University Third Hospital Beijing China; ^5^ PegBio Co., Ltd. Suzhou Jiangsu China

## Abstract

PB‐201 is the second glucokinase activator in the world to enter the phase III clinical trials for the treatment of type 2 diabetes mellitus (T2DM). Combined with the efficacy advantages and the friendly absorption, distribution, metabolism, and excretion characteristics, the indication population of PB‐201 will be broad. Because the liver is the primary organ for PB‐201 elimination, and the elderly account for 20% of patients with T2DM, it is essential to estimate PB‐201 exposure in specific populations to understand the pharmacokinetic characteristics and avoid hypoglycemia. Despite the limited contribution of CYP3A4 to PB‐201 metabolism in vivo, the dual effects of nonspecific inhibitors/inducers on PB‐201 (substrate for CYP3A4 and CYP2C9 isoenzymes) exposure under fasted and fed states also need to be evaluated to understand potential risks of combination therapy. To grasp the unknown information, the physiologically‐based pharmacokinetic (PBPK) model was first developed and the influence of internal and external factors on PB‐201 exposure was evaluated. Results are shown that the predictive performance of the mechanistic PBPK model meets the predefined criteria, and can accurately capture the absorption and disposition characteristics. Impaired liver function and age‐induced changes in physiological factors may significantly increase the exposure under fasted state by 36%–158% and 48%–82%, respectively. The nonspecific inhibitor (fluconazole) and inducer (rifampicin) may separately increase/decrease PB‐201 systemic exposure by 44% and 58% under fasted state, and by 78% and 47% under fed state. Therefore, the influence of internal and external factors on PB‐201 exposure deserves attention, and the precision dose can be informed in future clinical studies based on the predicted results.

**Study Highlights**

**WHAT IS THE CURRENT KNOWLEDGE ON THE TOPIC?**

CYP3A4 (metabolism fraction [*f*_m_]‐36.0%) and CYP2C9 (*f*_m_‐32.7%) are the major isoenzymes of PB‐201 metabolism in vitro, and food can significantly increase PB‐201 exposure in vivo. Clinical drug‐drug interaction studies have shown that CYP3A4 has the limited (~26.4%) contribution to PB‐201 elimination in vivo, indicating that the *f*_m_ of CYP2C9 in vivo may be less than 25%.

**WHAT QUESTION DID THIS STUDY ADDRESS?**

Dual effects of nonspecific inhibitors/inducers on PB‐201 exposure under fasted and fed states were evaluated by the physiologically‐based pharmacokinetic model to understand potential risks of combination therapy. Meanwhile, the exposure in specific populations was also evaluated to provide supportive information for corresponding clinical trials.

**WHAT DOES THIS STUDY ADD TO OUR KNOWLEDGE?**

The nonspecific inhibitor (fluconazole) and inducer (rifampicin) may separately increase/decrease PB‐201 systemic exposure by 44% and 58% under fasted state, and by 78% and 47% under fed state. Physiological changes due to impaired hepatic function and age may significantly increase exposure under fasted state by 36%–158% and 48%–82%, respectively.

**HOW MIGHT THIS CHANGE DRUG DISCOVERY, DEVELOPMENT, AND/OR THERAPEUTICS?**

Combined with the exposure‐response analysis results, the effects of nonspecific inhibitors may lead to hypoglycemia in some patients and the nonspecific inducers can significantly reduce PB‐201 exposure under fasted and fed states. But the predicted results still need to be confirmed in future clinical trials. Assuming that the same systemic exposure of PB‐201 produces the same pharmacological effects in vivo, the predicted results can be served as indicators for the dose design of corresponding clinical trials.

## INTRODUCTION

Glucokinase (GK), as a glucose sensor in the β‐cells of the pancreas and a pacemaker in the hepatic conversion of glucose to glycogen,^1^ is considered to be the major rate‐limiting enzyme for glycolysis.^2^ Small‐molecule GK activators (GKAs) have overcome physical limitations to effectively control the levels of glycosylated hemoglobin in patients with type 2 diabetes mellitus (T2DM)^3^, ^4^ and have the potential to reverse diabetes.^5^ Currently, a similar drug has successfully reduced glucose^6^ and improved the early insulin secretion index in clinical trials,^7^ indicating that GKA will play a unique role in hypoglycemic drugs. As the second GKA in the world to be developed in the phase III clinical trials,^8^ PB‐201 (imported from Pfizer [PF‐04937319] by Suzhou PegBio Co., Ltd.) has demonstrated safe^9^, ^10^, ^11^ and effective hypoglycemic effects^11^ in various early clinical trials.^9^, ^10^, ^11^

Currently, a series of pharmacokinetic (PK) studies of PB‐201 tablets in vivo have been completed. The main PK characteristics of PB‐201 in vivo indicate that PB‐201 has the moderate oral bioavailability (about 68%, the detailed data unpublished) in monkeys.^12^ PB‐201 is slowly absorbed in humans, with the time (median) to reach maximum plasma concentration after multiple doses (NCT01272804) about 2–3 h. The binding rate of PB‐201 to plasma proteins is about 70%,^13^ suggesting that PB‐201 is widely distributed in vivo (volume of distribution based on the terminal phase ~254.4 L at 100 mg, NCT01044537). Cytochrome P450‐mediated oxidative metabolism plays the major role in the elimination of PB‐201, which was catalyzed through CYP3A and CYP2C9 isoforms.^13^ Moreover, PB‐201 is not a specific substrate of OATP1B1, OATP1B3, P‐gp, and BCRP (unpublished). The kidney contributes a little to PB‐201 elimination in vivo, as unchanged drug in the urine is less than ~1% (NCT01272804). Biliary excretion has a tiny (about 2.3%) contribution to the elimination of PB‐201 in the bile duct‐cannulated rats.^12^ Therefore, the liver plays an important role in the clearance of PB‐201, and evaluation of the systemic exposure of PB‐201 in the liver impairment population is necessary and pressing in the development of new drugs.^14^

Globally, there are about 536.6 million people aged 20–79 years with diabetes, of whom more than 90% suffer from T2DM,^15^ and the elderly account for 20% of patients with T2DM.^16^ Generally, drug safety is closely related to the systemic exposure.^17^ Specific populations, including the liver impairment population, geriatric population, and so on, usually have increased^18^ or delayed absorption,^19^ abnormal enzyme activity,^20^, ^21^, ^22^ and changes in other physiological factors, which will cause uncertain changes in systemic exposure and induce adverse effects. Thus, assessment of PB‐201 systemic exposure in specific populations is the prerequisite for avoiding hypoglycemic events before expanding the clinical indication population. However, the disposition mechanism of PB‐201 cannot be summarized based on the PK characteristics described above, and the lack of understanding of the disposition mechanism will increase the difficulty of reasonably designing the dose of PB‐201 in the clinical trials with specific populations. Moreover, the improper dosage may also induce hypoglycemia. Therefore, recommending scientific and reasonable doses in clinical trials for specific populations has become the crucial step to ensure the safe and effective implementation of clinical trials.

Up to now, a drug–drug interaction (DDI) study of PB‐201 has only been conducted with ketoconazole (a specific strong CYP3A4 inhibitor), which has minimal effect on the systemic exposure of PB‐201 (about 26.4%). Due to the limited contribution of CYP3A4 to systemic exposure of PB‐201 and extensive metabolism of PB‐201 by multiple isoenzymes, the induction of perpetrators on PB‐201 exposure has not been evaluated yet. Although CYP2C9 is the secondary CYP isoform for the clearance of PB‐201 in vitro, the activity of CYP2C9 can also be inhibited by fluconazole and fluvoxamine,^23^ which are also CYP3A4 inhibitors. Compared with the effect of ketoconazole on PB‐201 systemic exposure, whether fluconazole and fluvoxamine have similar or intensive effects is worth discussing. Moreover, the systemic exposure of PB‐201 was significantly increased under fed state (NCT01513928) compared with the same dose of PB‐201 under fasted state (NCT01272804). In order to avoid the treatment failure in phase III studies, there is an urgent need to fully understand the systemic exposure of PB‐201 co‐administration with perpetrators both under fasted and fed states, so as to provide supportive information for dose decision in the phase III clinical trials.

Considering that the physiologically‐based pharmacokinetic (PBPK) model can bridge the relationship between in vitro experimental results and in vivo systemic exposure,^24^, ^25^ evaluation of PB‐201 co‐administration with perpetrators can be performed through the model method after the disposition mechanism of compound validated by DDI studies with strong index perpetrators.^26^ Meanwhile, untested scenarios can be simulated based on the population library to explore the effects of external and internal factors on the PB‐201 systemic exposure.^27^ Herein, we are aimed (i) to develop a mechanistic PBPK model of PB‐201 according to preclinical and clinical data to reliably describe the absorption and disposition characteristics of PB‐201 in vivo; (ii) to explore the influences of physiological changes on the systemic exposure of PB‐201 to support dose decision in clinical trials of specific populations; and (iii) to evaluate the changes in systemic exposure after PB‐201 co‐administration with potential perpetrators under both fasted and fed states to provide useful information for ensuring the successful implementation of PB‐201 phase III clinical trials.

## MATERIALS AND METHODS

### Study strategy

**FIGURE 1:**
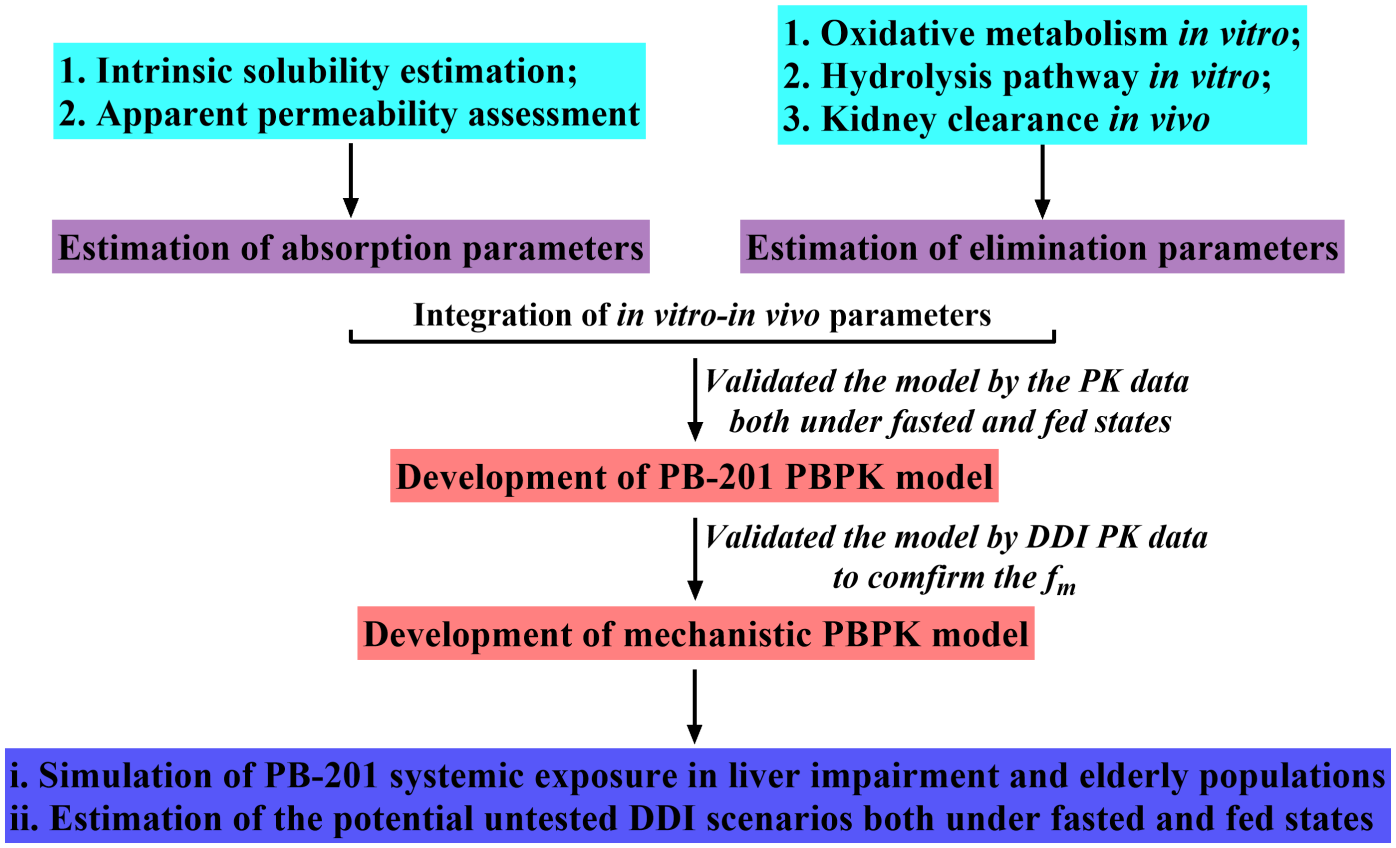
Workflow for the development and application of PB‐201 PBPK model. DDI, drug‐drug interaction; *f*_m_, metabolism fraction; PBPK, physiologically‐based pharmacokinetic; PK, pharmacokinetic.

PB‐201 PBPK model was developed according to the in vitro–in vivo data, such as the permeability and oxidative metabolism of PB‐201 in vitro and the renal clearance of PB‐201 in humans. Then, the model was validated by the clinical trial results including PK studies results of multiple ascending doses both in White and Chinese patients (NCT01272804 and NCT03973515), PK study results of PB‐201 with a single‐dose immediate release formation under fed state (NCT01513928), PK study results of PB‐201 in adults with T2MD inadequately controlled on metformin (NCT02206607), and the DDI study results about ketoconazole co‐administration with PB‐201 (NCT01468714). Finally, the mechanistic PBPK model was used to simulate the PK characteristics of PB‐201 in specific populations and to evaluate the effects of CYP3A/CYP2C9 perpetrators on PB‐201 systemic exposure both under fasted and fed states in order to provide supportive information for dose decision making in corresponding clinical trials. The detailed strategy diagram is shown in Figure 1, the simulation scenarios are displayed in Table S1, and the perpetrators' model parameters are listed in Tables S2 of the supplementary files.

### Apparent permeability and P‐gp substrate assessment in Caco‐2 cell lines

The integrated Caco‐2 monolayers were cultured for 22 days in a 5% CO_2_ incubator at 37°C for the assessment of PB‐201 apparent permeability. Monolayers were incubated with PB‐201 in apical and basolateral (BL) sides for 2 h at the concentration of 0.1 μM, 1 μM, and 5 μM, respectively. Meanwhile, nadolol (with low permeability compound), propranolol (with high permeability), and taxol (P‐gp substrate) were selected as the positive controls to ensure the applicability of the incubation system. Moreover, verapamil, a specific inhibitor of P‐glycoprotein (P‐gp), was added into another incubation system under the same condition mentioned above to evaluate whether PB‐201 was a substrate of P‐gp. All experiments were conducted in triplicate and analyzed by high performance liquid chromatography–tandem mass spectrometry (HPLC‐MS/MS). The apparent permeability coefficient (*P*_app_) was calculated based on the following equation:

(1) $$P_{\mathrm{app}}=\frac{dQ}{dt}\times \frac{1}{A\times C_{0}}$$

where *P*_app_ was cm/s × 10^−6^, d*Q*/d*t* (pmol/second) was the rate at which the compound appears in the receiver side, *C*_0_ (nM) was the initial concentration of the compound in the donor side, and *A* (cm^2^) represents the surface area of the cell monolayer.

### Metabolic stability of PB‐201 in human recombinant CYP isoenzymes

Due to CYP‐mediated oxidative metabolism was the main route for the elimination of PB‐201 in preclinical animal studies,^12^ human recombinant CYP1A2, CYP2B6, CYP2C8, CYP2C9, CYP2C19, CYP2D6, and CYP3A4 isoenzymes were selected to evaluate the metabolic stability of each isoenzyme to PB‐201. The 100 μM PB‐201 with the volume of 2 μL was added into the 100 μL CYPs (100 pmol/mL) working solution. After gently mixing, the mixture was pre‐incubated at 37°C for 10 min. The reaction was initiated by adding 98 μL of cofactor working solution. Samples were collected at 0, 5, 15, 30, and 60 min, respectively, and the reaction was terminated with cold acetonitrile. After vortexing vigorously for about 1 min, samples were centrifuged at 3000 *g* for 15 min at 4°C. PB‐201 in the supernatant was removed and quantified by HPLC‐MS/MS. Moreover, to ensure the robustness of the incubation system, the metabolic stability of positive control compounds of corresponding CYP isoenzymes was evaluated by the same method. The intrinsic clearance (CL_int_) of each CYP isoenzymes to the metabolism of PB‐201 was calculated according to the remaining percentage of PB‐201 and the amount of CYP isoform in the incubation system. The detailed equation is listed in Equation 2. Moreover, according to the published protein abundance of each CYP isoenzymes in the human liver,^28^ metabolism fraction (*f*_m_) of each CYP isoenzyme for PB‐201 systemic metabolism in vitro was calculated based on Equation 3.^29^

(2) $$\begin{array}{c} {\mathrm{CL}}_{\mathrm{int}\;P450j}=-\text{slope of}\;\ln\left(\%\text{drug remaining}\right)\\ \;\mathrm{vs}\;\text{time}\times \frac{\text{volume of incubation}\;\left(\mathrm{uL}\right)}{\text{amount of}\;P450_{j}\;\left(\text{pmol}\right)} \end{array}$$

where CL_int *j*_ was *j*th CYP isoform and the P450_*j*_ was the *j*th P450 isoform tested.

(3) $$\%\text{contribution},P450_{J}=\frac{{\mathrm{CL}}_{\mathrm{int}\;P450j}\times P450_{j}\text{abundance}}{\sum_{j=1}^{n}{\mathrm{CL}}_{\mathrm{int}\;P450j}\times P450_{j}\text{abundance}}$$

where P450_*j*_ abundance was the protein abundance of P450_*j*_.

### The development of PB‐201 PBPK model

The PB‐201 PBPK absorption model was developed by the advanced dissolution, absorption, and metabolism model in SimCYP Population‐Based Simulator (version 19; SimCYP Limited, Sheffield, UK, Certara Company). The Caco‐2 Transwell model coupled with the diffusion layer model (DLM) were used to describe the absorption characteristics of PB‐201. Meanwhile, the *P*_app_ of PB‐201 was calibrated based on the positive control group (propranolol, one of the model drugs embedded in SimCYP) through the permeability calibrator embedded in the prediction toolbox of the SimCYP simulator.^30^ Due to the limited in vitro experimental results to support the development of DLM, all parameters in the DLM were default values except the intrinsic solubility (*S*_*o*_), which was obtained through sensitivity analysis and fitting the PK data in clinical trials. The full‐PBPK model with the predicted steady‐state distribution volume and tissue to plasma partition coefficient (*K*_p_) was used to describe the distribution characteristics of PB‐201 in vivo. Moreover, *K*_p_ scalar was estimated to be 1.50 to match the observed concentration‐time profiles. Because the metabolic profile in human hepatocytes revealed that PB‐201 was metabolized through oxidative (major) and hydrolytic pathways (minor),^13^ the oxidative pathway of PB‐201 in vivo was characterized by the CL_int_ of the corresponding CYP isoenzyme according to the metabolic stability studies in human recombinant incubation systems and the hydrolytic pathway of PB‐201 in vivo was characterized by CL_int_Hep_ of hepatocytes. Meanwhile, renal clearance (CL_R_) obtained from a clinical trial was used to represent the unchanged PB‐201 in urine. Because the intrinsic clearance of PB‐201 hydrolysis in hepatocytes was absent and the contribution percentage of CYP3A4 to the PB‐201metabolism in vivo had been confirmed in the DDI study (NCT01468714), CL_int_Hep_ and inter‐system extrapolation factors (ISEFs) scaling of recombinant CYP isoforms in vitro kinetic data were fitted according to the contribution of CYP isoforms in vitro and in vivo (NCT01468714). Last but not least, physicochemical parameters including molecular weight, protein binding, B/P ratio, Log *P*, and *pK*_a_ were obtained from experimental outcomes.

### Validation of the PBPK model

Validation of PB‐201 PBPK model was based on five clinical studies, including multiple ascending dose (MAD) studies in White volunteers (NCT01272804) and Chinese (NCT03973515) volunteers, the PK study of PB‐201 immediate release solid formulation in overweight and obese otherwise healthy volunteers under fed state (NCT01513928), the PK study about PB‐201 in adult patients with T2MD inadequately controlled on metformin (NCT02206607), and the DDI study of PB‐201 co‐administration with ketoconazole (NCT01468714). The trial design in the SimCYP simulator was consistent with the corresponding dosage regimen and blood sampling time points. Furthermore, all simulation clinical trials, including population demographics (age, sex, and ethnicity), were conducted with a virtual population (10 trials with 10 subjects, *n* = 100). Above all, the predictive performance of the PB‐201 PBPK model was comprehensively estimated with two criteria: (i) the observed concentration‐time profile was within the 90% confidence interval (CI) of predicted ones; and (ii) the ratio of major PK parameters (area under the curve [AUC] and maximum plasma concentration [*C*_max_]) was within a predefined boundary of 0.5–2.0 folds.

### Pharmacokinetic simulations

Because the hypoglycemic effect of PB‐201 was related to the systemic exposure within a certain dose range (unpublished), we assumed that the same systemic exposure of PB‐201 would produce the same pharmacological effect in vivo. Accordingly, the validated PB‐201 PBPK model was used to simulate untested scenarios. “Healthy Volunteer,” “Chinese Healthy Volunteer,”, specific populations in the default population database, and the PBPK model of perpetrators provided by SimCYP software were directly used to simulate the systemic exposure of PB‐201 under regimen A (100 mg in the morning and noon) in different scenarios except for special announcement.

#### Simulation of PB‐201 in specific populations

Systemic exposure of PB‐201 in specific populations, including liver impairment and geriatric populations, were simulated based on SimCYP physiological model. Because the parent drug was scarcely excreted through the kidneys in the clinical study (NCT01272804), the systemic exposure of PB‐201 in the renal impairment population was not simulated. To accurately evaluate the effects of physiological factors on systemic exposure, liver impairment population was divided into three subsets according to the Child‐Pugh scores A, B, and C, which were corresponded to mild, moderate, and severe liver impairment, respectively. Meanwhile, the geriatric population contained two groups: people aged 65–75 and 75–85 years. All simulations were performed under the predesigned scenarios listed in Table S1A, and the effects of physiological factors on PB‐201 systemic exposure were quantitatively evaluated by comparing the major PK parameters (AUC from time point 0 to the end of the dosing interval [AUC_0–*t*_] and C_max_) in specific populations with those in the “Healthy Volunteer” group.

#### Estimation of the potential effects of perpetrators on PB‐201 systemic exposure

Untested clinical DDI scenarios, such as PB‐201 concomitant administration with strong CYP3A inhibitor (itraconazole), moderate CYP3A inhibitor (erythromycin), mild CYP3A inhibitor (cimetidine), moderate inducer (efavirenz), mixed‐type inhibitor/inducer fluconazole (CYP3A and CY2C9 moderate inhibitor), fluvoxamine (CYP3A moderate inhibitor and CYP2C9 mild inhibitor), and rifampicin (CYP3A strong inducer and CYP2C9 moderate inducer) were simulated under both fasted and fed states. To obtain the maximum effect of perpetrator on PB‐201 systemic exposure, perpetrator was given daily at the maximum clinical dosage regimen until the end of the simulation, and PB‐201 was given when the steady‐state concentration of perpetrator was reached. The detailed regimens about DDI simulations are summarized in Table S1B.

## RESULTS

### Apparent permeability and the P‐gp specific substrate assessment

The intact Caco‐2 cell monolayers were utilized for PB‐201 apparent permeability and specific substrate evaluation. The *P*_app_ values of PB‐201 at the concentration of 0.1, 1, and 5 μM were 9.57, 9.33, and 7.75 × 10^−6^ cm/s, respectively, indicating that PB‐201 was a compound with medium permeability. Although the efflux ratio (ER) of PB‐201 was decreased by 49.99% when incubated with verapamil (P‐gp specific inhibitor) in BL, the ER was less than 1 in the absence of verapamil. Therefore, PB‐201 is not a specific substrate of P‐gp. The detailed results are shown in Table 1.

**TABLE 1:** Apparent permeability of each test compound in Caco‐2 monolayers.

Compounds	Concentration (μM)	*P*_app,AP→BL_ (10^−6^ cm/s)		*P*_app,BL→AP_ (10^−6^ cm/s)		Efflux ratio *P*_app,BL→AP/AP→BL_	
		Without inhibitor	With inhibitor	Without inhibitor	With inhibitor	Without inhibitor	With inhibitor
Nadolol	10	0.721	0.657	0.634	0.364	0.880	0.554
Propranolol	10	29.4	57.4	23.1	21.9	0.785	0.382
Taxol	10	0.0798	1.17	0.56	1.28	7.02	1.09
PB‐201	0.1	9.57	11.8	7.94	5.93	0.830	0.502
	1	9.33	10.2	6.31	4.77	0.676	0.468
	5	7.75	10.5	6.14	5.58	0.792	0.530

Abbreviations: AP, apical; BL, basolateral; *P*_app_, apparent permeability coefficient.

### The metabolic stability of PB‐201 in human recombinant CYP isoenzymes

The oxidative metabolic stability of PB‐201 in the liver was evaluated in human recombinant CYP1A2, CYP2B6, CYP2C8, CYP2C9, CYP2C19, CYP2D6, and CYP3A4 isoenzymes. All isoenzymes were involved into the metabolism of PB‐201, and the remaining percentage of PB‐201 in the different incubation systems over time is presented in Figure S1 of the supplementary file. The CL_int_ of each CYP isoenzyme to PB‐201 metabolism was calculated according to Equation 2, as shown in Table 2. Meanwhile, the *f*_m_ of each isoenzyme to PB‐201 metabolism in vitro was obtained based on the protein abundance of each CYP isoform in the human liver, where CYP3A4 (about 36.0%) and CYP2C9 (about 32.7%) were the main CYP isoenzymes of PB‐201 metabolism in vitro.

**TABLE 2:** Intrinsic clearance and metabolism fraction of PB‐201 in various CYP isoenzymes.

CYP P450 isoform	Mean specific CYP content (pmol CYP/mg)	CL_int_ (μL/min/pmol)	*f*_m_ (%)
CYP1A2	45	0.068	7.20
CYP2B6	39	0.072	6.60
CYP2C8	64	0.060	9.05
CYP2C9	96	0.144	32.7
CYP2C19	19	0.113	5.05
CYP2D6	10	0.145	3.42
CYP3A4	108	0.141	36.0

Abbreviations: CL_int_, intrinsic clearance; *f*_m_, metabolism fraction.

### PBPK model validation

**FIGURE 2:**
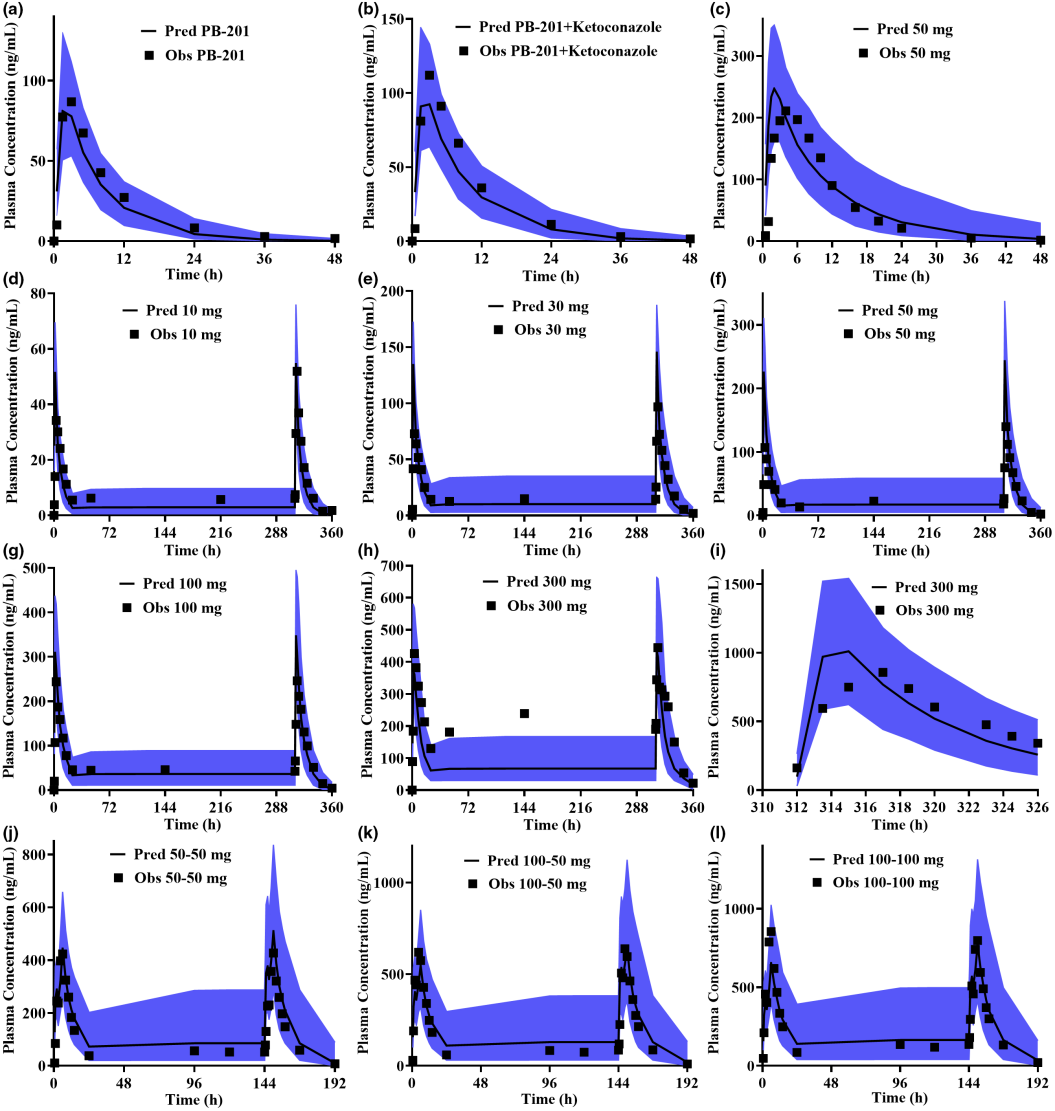
The validated results for PB‐201 PBPK model. (a, b) Vvalidated by the DDI study in White patients; (c) was validated with the PK data under fed state; (d–h) were validated by the clinical data of MAD studies in White patients; (i) was validated with the clinical study of PB‐201 in patients with T2DM; (j–l) were validated by the clinical dada of MAD studies in Chinese patients. The blue range is the 90% confidence interval of the predicted results. DDI, drug‐drug interaction; MAD, multiple ascending dose; Obs, observed value; PK, pharmacokinetic; Pred, predicted value.

**FIGURE 3:**
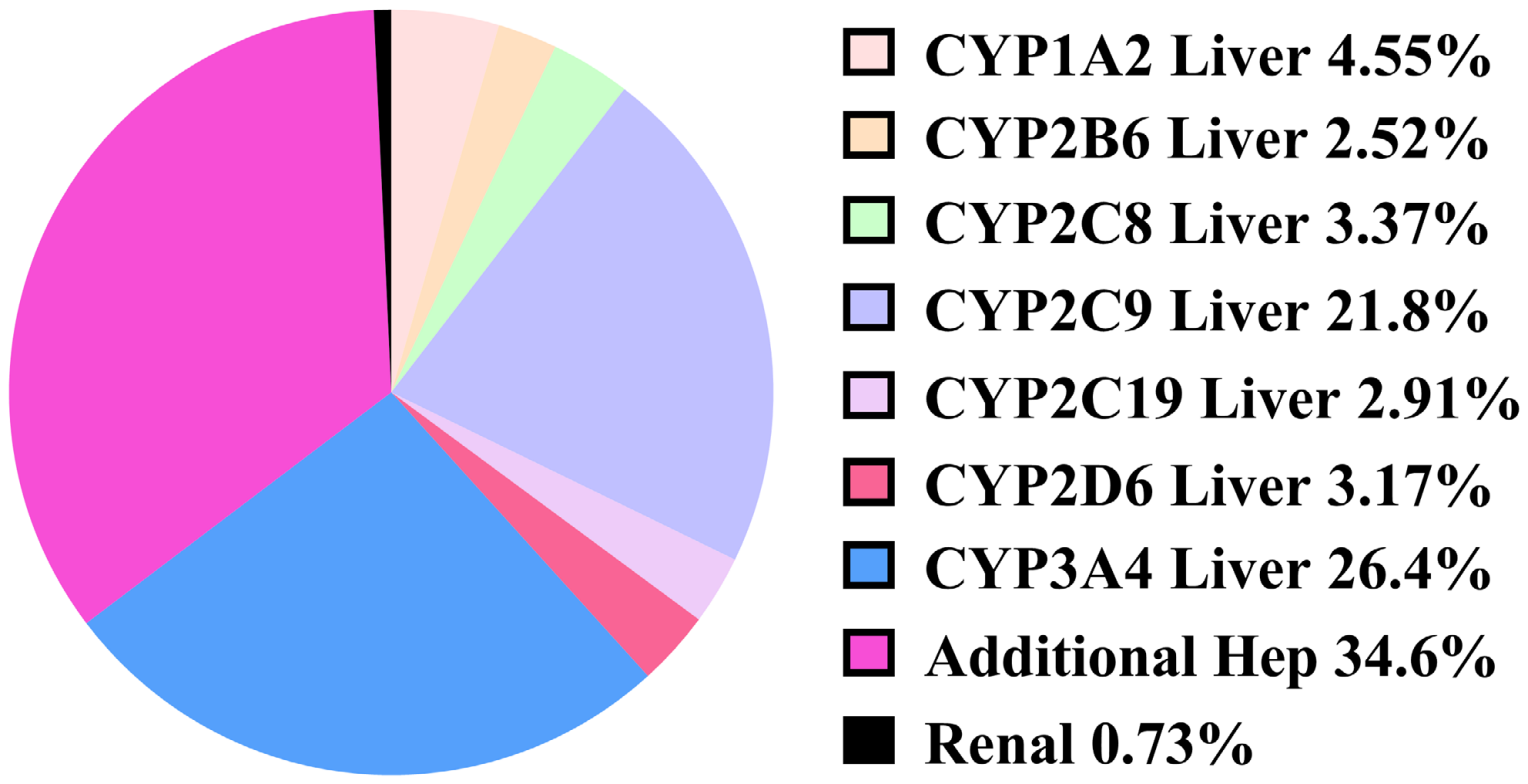
The contribution percentage of each organ/isoenzyme to the disposition of PB‐201 under regimen A in Chinese volunteers. Hep, hepatocyte.

The PB‐201 PBPK model was validated by several clinical data, and the predictive performance was satisfactory. The predicted systemic exposure of PB‐201 in the absence or presence of ketoconazole was in good agreement with the observed results (Figure 2a,b), suggesting that the metabolic mechanism of PB‐201 was well‐captured by the model. Meanwhile, the absorption phase of PB‐201 under fed (Figure 2c) and fasted states (other picture in Figure 2) matched well, indicating that the model had the ability to capture absorption characteristics. Moreover, the 90% CI of the predicted plasma concentration‐time curve included the observed concentration‐time points (Figure 2), and the major PK parameters were within the predefined boundaries (Figure S2). Accordingly, the PBPK model was able to balance the absorption and disposition characteristics of PB‐201 in vivo. On the basis of the validated CYP3A4 metabolic pathway, the amount of unchanged PB‐201 in urine and the systemic metabolism of PB‐201 in the human recombinant CYP isoform incubation system, the contribution percentage of each organ/isoenzyme to the elimination of PB‐201 in Chinese patients under regimen A was calculated in SimCYP and presented in Figure 3. The final PB‐201 PBPK model parameters and data sources are summarized in Table S3 of the supplementary file.

### Evaluation of the effects of internal and external factors on PB‐201 systemic exposure

#### Evaluation of PB‐201 systemic exposure in specific populations

**FIGURE 4:**
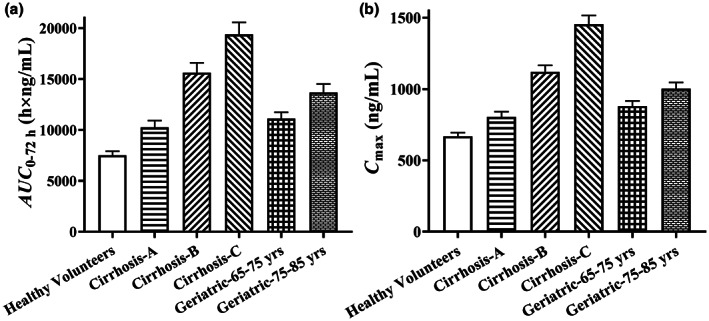
The predicted systemic exposure of PB‐201 in specific populations under fasted state. AUC_0‐72 h_, area under the curve from zero to 72 h; *C*_max_, maximum plasma concentration.

Liver is the predominant elimination organ of PB‐201. Thus, systemic exposure of PB‐201 under fasted state was increased by 36%, 108%, and 158% in patients with mild, moderate, and severe liver impairment, respectively. The geriatric population with weak metabolic capacity may have a higher exposure level,^20^ as the AUC from zero to 96 h (AUC_0–96 h_) increases with age (48% and 82% for 65–75 and 75–85 years old, respectively; Figure 4). Considering that PB‐201 is safe and tolerable in the dose range of 3–640 mg in clinical studies and the overall total incidence of hypoglycemia is about 3% (exposure‐response relationship, unpublished), the predicted results can be served as an indicator for dose adjustment in future clinical trials.

#### Evaluation of the potential DDI effects on PB‐201 systemic exposure

**FIGURE 5:**
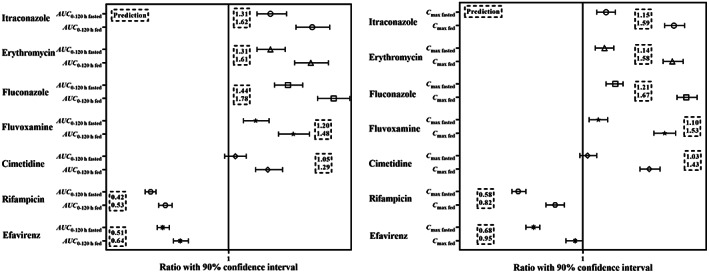
The predicted changes in systemic exposure after PB‐201 co‐administration with potential perpetrators both under fasted and fed states. AUC_0‐120 h_, area under the curve from zero to 120 h; *C*_max_, maximum plasma concentration.

The potential DDI scenarios were simulated in “Chinese Healthy Volunteers” with the mechanistic PB‐201 PBPK model. Because the contribution percentage of CYP3A4 to the clearance of PB‐201 was about 26.4% in vivo under fasted state, the specific CYP3A4 inhibitor itraconazole had limited effect on PB‐201 systemic exposure both under fasted (31%) and fed (62%) states (Figure 5). Fluconazole/fluvoxamine could simultaneously inhibit the activity of CYP3A4 and CYP2C9 isoforms, and increased AUC from zero to 120 h (AUC_0–120 h_) by 44%/20% and 78%/48% under fasted and fed states, respectively. Cimetidine, the mild CYP3A4 inhibitor, had a weak effect on PB‐201 systemic exposure, increasing AUC_0–120 h_ by 5% under fasted state and 29% under fed state. Moderate CYP3A4 inducer had the noticeable influence on PB‐201 systemic exposure, and the AUC_0–120 h_ was decreased by 49% under fasted state and 36% under fed state. Rifampin, a CYP3A strong inducer and CYP2C9 moderate inducer, could significantly enhance the enzyme activity, resulting in a decrease of AUC_0–120 h_ by 58% and 47% under fasted and fed states, respectively. Although erythromycin is a moderate CYP3A inhibitor, the time‐dependent inhibition of erythromycin has a powerful effect on CYP3A isoenzyme.^31^ Compared with CYP3A4 strong inhibitor itraconazole, erythromycin had a similar impact on PB‐201 systemic exposure (AUC_0–120 h_ increased by 31% and 61% under fasted and fed states, respectively). All predicted results are presented in Figure 5.

## DISCUSSION

Absorption is a complex process that can be affected by formulation factors, physiological parameters, food composition, and so on. As the weakly basic insoluble oral immediate release solid formulation with moderate permeability, dissolution in the gastrointestinal tract is the first and pivotal step for PB‐201 to enter into the systemic circulation. The limited experimental data available for the development of PB‐201 absorption model (in 2020) include only the solubility of the active pharmaceutical ingredient and dissolution profiles of formulations in the aqueous buffer media, which cannot represent dissolution behavior of PB‐201 in the gastrointestinal tract, resulting in the failure to capture absorption characteristics of PB‐201 in vivo by PBPK model. To match the absorption phase of PB‐201 in the concentration‐time profiles obtained from multiple clinical trials, a diffusion layer model was used in the absorption‐formulation module, which was developed by adjusting the value of *S*_*o*_ in the model according to sensitivity analysis. Although other parameters used in the DLM were default values, the sensitivity analysis showed that these parameters had no significant influence on PB‐201 systemic exposure. Notably, based on the later (in 2023) obtained solubility of PB‐201 in various aqueous and biorelevant media, SIVA software evaluated intrinsic solubility of PB‐201 was about 0.003 mg/mL and the log *K*_m:w, neutral/ion_ was about 1.00E‐06. When the absorption model was developed based on the in vitro experimental results, the distribution and metabolism parameters were kept consistent with the PBPK model presented in this paper. Considering that the dissolution of tablets in vivo is a function of several factors and other parameters in PB‐201 model are default values in SimCYP, the value of *S*_*o*_ (0.000123 mg/mL) obtained based on the sensitive analysis is used in the model to balance the overestimated default values, such as the log *K*_m:w, neutral/ion_. Moreover, the *K*_*p*_ scalar for the volume of distribution was adjusted to match the PK profile, but the value in this paper was the optimal solution, which was obtained by performing a sensitivity analysis both *S*_*o*_ and *K*_*p*_. Furthermore, there were no significant species differences among rats, dogs, and monkeys because the allometric exponent (b) in rats, dogs, and monkeys was about 0.74, which was calculated based on the clearance (intravenous administration), body weight, and the plasma unbound fraction of PB‐201 in corresponding species. Assuming similar bioavailability in humans and monkeys (68% in monkeys, unpublished) and an average body weight of ~76.89 kg in humans (the mean value of simulated healthy volunteers in SimCYP), the predicted and observed distribution volume of PB‐201 in humans was also compared. The ratio of the predicted value (1.884 L/kg) to the observed one (1.983 L/kg) was about 0.95. Therefore, the predicted volume of distribution is acceptable. Additionally, CL_int_, CL_int_hep_, and CL_R_ were included in the model to match the elimination characteristics of PB‐201 with the method of in vitro*–*in vivo extrapolation. Because the systemic exposure of PB‐201 was captured both under fasted and fed states, and the elimination characteristics were validated by the DDI study of PB‐201 co‐administration with strong CYP3A4 inhibitor and the values of systemic clearance, absorption and disposition characteristics of PB‐201 in vivo were well balanced and captured by the PBPK model. With the good predictive performance of the PB‐201 PBPK model in the validation of multiple clinical trials, the current PBPK model still has the reliable predictive ability for assessing untested scenarios (DDI and PK in specific populations), although the absorption model parameters are obtained by the method of “fit‐for purpose.”

Generally, there exist a positive bidirectional association between T2DM and nonalcoholic fatty liver disease,^32^, ^33^ and the elderly account for about 20% of the total number of patients with diabetes.^16^ The impaired liver function and physiological changes in patients with T2DM may be the key factors leading to the interindividual variation of PB‐201 in vivo. Hence, simulation of PB‐201 in liver impairment and geriatric populations is the crucial link to understand the potential effect of internal factors on systemic exposure and the necessary step to broaden the application groups.^14^ However, the PBPK model may have a minor drawback in predicting the hepatic metabolic mechanism. Because the hydrolysis pathway in the hepatocytes was compensated by the “fit‐for‐purpose” method, and the value of CL_int_Hep_ was obtained by fitting the PK profiles of PB‐201 in the absence/presence of ketoconazole under the condition of fixing the CL_int_ of each isoform (NCT01468714). Moreover, according to the percentage of remaining PB‐201 in the incubation system and the protein abundance in human liver,^28^ the *f*_m_ calculated in this paper was significantly different from previously published results calculated based on the generation of metabolites (M1).^13^ Excluding the contribution of nonoxidative pathways (about 35.3%) to PB‐201 elimination in vivo, the *f*_m_ of CYP3A4 in vitro calibrated according to the overall contribution percentage of oxidative metabolism (about 64.7%) in vivo was about 23.3%, which was close to the *f*_m_ of CYP3A4 mediated PB‐201 elimination in vivo (26.4%). Therefore, the metabolic stability of PB‐201 in human recombinant CYP isoenzymes obtained in this paper was used to characterize the oxidative metabolic characteristics of PB‐201 in vivo. Meanwhile, according to the calibrated *f*_m_ in vitro, the ISEF was used to adjust the *f*_m_ of each CYP isoform to the elimination of PB‐201 in vivo. Although just the CYP3A4 pathway was confirmed by a DDI study in vivo, the predicted systemic clearance was consistent with the observed one and the liver metabolism was the main pathway to the elimination of PB‐201 in the model. Therefore, the predicted systemic exposure of PB‐201 in the specific populations can serve as an indicator for dose decision making in corresponding clinical trial designs.

PB‐201 was eliminated through various cytochrome oxidases, among which CYP3A4 and CYP2C9 were the main isoenzymes of PB‐201 metabolism, because the *f*_m_ of CYP3A4 and CYP2C9 was 36.0% and 32.7%, respectively, among the isoenzymes investigated in this paper. Due to the PB‐201 systemic exposure was slightly increased (about 26.4%) after co‐administration with the strong CYP3A inhibitor in the DDI clinical study (NCT01468714), the *f*_m_ of CYP2C9 to PB‐201 metabolism in vivo might be less than 25%. Therefore, the DDI study in vivo did not further evaluate the effect of CYP2C9 perpetrators on PB‐201 systemic exposure. Although ketoconazole could simultaneously inhibit the activities of CYP3A and CYP2C9 in SimCYP, the *K*_*i*_ value (10 μM; Table S2) of ketoconazole against CYP2C9 was greater than the maximum concentration of ketoconazole in the liver (8.63 μM). Therefore, ketoconazole has no positive potential to inhibit CYP2C9‐mediated metabolic pathways during simulation. However, other potential DDI scenarios need to be simulated by PBPK model, as some CYP3A4 inhibitors/inducers can simultaneously impact the activity of CYP3A and CYP2C9 in vivo. Under the preset scenarios, fluconazole (moderate CYP3A and CYP2C9 inhibitors) increased PB‐201 systemic exposure in adults aged 20–50 years by 44% under fasted state and 78% under fed state, which were the greatest influence of the specific inhibitors mentioned in this paper both under fasted and fed stated, respectively. But the predicted systemic exposure (AUC_0–120 h_ and C_max_) was still within the range of exposure‐response analysis (unpublished). With the assumption that the same systemic exposure of PB‐201 will produce the same pharmacological effect in vivo, PB‐201 co‐administration with specific inhibitors mentioned in this paper under both fasted and fed states may lead to hypoglycemia in some patients according to the exposure‐response analysis results (unpublished). Considering that the CYP2C9 pathway has not been validated, it is better to evaluate the potential effect of fluconazole on PB‐201 in clinical study. Additionally, rifampin (CYP3A strong inducer and CYP2C9 moderate inducer) and efavirenz (moderate CYP3A inducer) could significantly reduce PB‐201 systemic exposure both under fasted and fed states. Considering that PB‐201 is not a specific substrate of P‐gp and the bioavailability of PB‐201 in monkeys is moderate,^12^ rifampin will not produce complex induction during absorption and metabolism.^27^ Hence, the predicted results about the rifampin and efavirenz induction can be used as the reference, but still need to be confirmed in future clinical trials.

Chronic kidney disease is a common complication of T2DM, affecting 50% of the worldwide patients with T2DM.^34^ Renal failure can reduce CL_R_, which in turn affects the activities of transporters and metabolic enzymes in the liver and gastrointestinal tract.^22^, ^35^ Therefore, the dosage of compounds eliminated through non‐renal transport and metabolism should also be adjusted in patients with the nephropathy.^35^ However, simulation in such a population was not performed in this study, as the parent drug of PB‐201 is scarcely secreted (<1%) by the kidneys. Furthermore, the renal impairment population in the SimCYP population library has not established the relationship between the changes of liver and gastrointestinal function and renal impairment. Therefore, the simulation results of PB‐201 in the renal impairment population cannot represent the systemic exposure in the patients with T2DM with chronic kidney disease, even if the simulation is performed. Accordingly, it is desirable to estimate PB‐201 systemic exposure in patients with T2DM complications of chronic kidney disease to manage unknown risks clinically^36^ and to expand the indication population in the future.

In retrospect, the PBPK model has become the favorable tool for applicants to understand untested scenarios in advance and then guide the clinical trial designs. Similarly, the mechanistic PB‐201 PBPK model allows a conservative evaluation of the possible scenarios. Due to the finite clinical data, simulations of PB‐201 in specific populations and co‐administration with an inducer have not been validated. Therefore, such predicted results can only serve as a reference for investigators/regulators to inform precision doses in a scientific and well‐founded manner. Additionally, because the liver impairment and geriatric populations in the SimCYP default population database are developed based on White patients,^37^ and there is no racial difference in PB‐201 exposure (unpublished data of population PKs), the predicted results in specific populations can also be used as indicators for future clinical trials in China.

## AUTHOR CONTRIBUTIONS

M.Z. wrote the manuscript. D.L., M.X., and H.L. designed the research. M.Z., Z.L., Z.Y., and X.Y. performed the research. M.Z. and Z.L. analyzed the data. D.L. contributed analytical tools.

## FUNDING INFORMATION

This work was supported by the Key Clinical Projects of Peking University Third Hospital (No. BYSY2018063) and Advancing Model‐Informed Drug Development and Regulation in China (No. INV‐007625).

## CONFLICT OF INTEREST STATEMENT

M.X. is employed by PegBio Co., Ltd. All other authors declared no competing interests for this work.

## Supporting information


Appendix S1
