# Supplementary material for: Development of a PBPK model to quantitatively understand absorption and disposition mechanism and support future clinical trials for PB‐201
Source: CPT Pharmacometrics Syst Pharmacol. 2023 Apr 20;12(7):941–52. doi: 10.1002/psp4.12964 (PMC10349193; doi:10.1002/psp4.12964)
Supplement: Supplementary file 1 — Appendix S1 [file PSP4-12-941-s001.docx]

**Tables:**

**Table S1** Summary of simulation scenarios in this manuscript

| 1. Simulation scenarios of specific populations with PB-201 100 mg at morning and noon under fasted state | | | |
| --- | --- | --- | --- |
| Healthy Volunteers (20-50 years old) | | | |
| Cirrhosis CP-A population (20-50 years old) | | | |
| Cirrhosis CP-B population (20-50 years old) | | | |
| Cirrhosis CP-C population (20-50 years old) | | | |
| Geriatric populations (65-75, and 75-85 years old) | | | |
| 1. Simulation scenarios of potential DDI in the Chinese under both fasted and fed states | | | |
| Perpetrators | Dosage regimens^[[1]](#footnote-1)^ | Treatment | PB-201  dosage regimens |
| Itraconazole  (Strong CYP3A inhibitor) | 200 mg QD | 13 | 100 mg at morning and noon (D9-D13) |
| Erythromycin  (Moderate CYP3A inhibitor) | 500 mg QID | 13 | 100 mg at morning and noon (D9-D13)) |
| Fluconazole  (Moderate CYP3A and CYP2C9 inhibitor) | 200 mg QD | 13 | 100 mg at morning and noon (D9-D13) |
| Fluvoxamine  (Mild CYP3A and CYP2C9 inhibitor) | 36.65 mg QD | 13 | 100 mg at morning and noon (D9-D13) |
| Cimetidine  (Mild CYP3A inhibitor) | 400 mg BID | 13 | 100 mg at morning and noon (D9-D13) |
| Rifampin  (Strong CYP3A and moderate CYP2C9 inducer) | 600 mg QD | 13 | 100 mg at morning and noon (D9-D13) |
| Efavirenz  (Moderate inducer) | 600 mg QD | 13 | 100 mg at morning and noon (D9-D13) |

**Table S2** Summary of the perpetrators’ model parameters (Obtained from SimCYP library with the version of 19)

| **Parameter** | **Input Value** | **Source** |
| --- | --- | --- |
| **Ketoconazole** | | |
| Physicochemical properties |  |  |
| Molecular weight (g/mol) | 531.4 |  |
| Log *P* | 4.04 |  |
| Compound type | Diprotic base |  |
| *pK*_a 1_ | 2.94 |  |
| *pK*_a 2_ | 6.51 |  |
| Blood-to-plasma partition ratio | 0.62 |  |
| Fraction unbound in plasma | 0.029 |  |
| Absorption |  |  |
| Absorption model | First-Order |  |
| User Input |  |  |
| fa/CV (%) | 1/30 |  |
| ka (1/h)/CV (%) | 0.78/30 |  |
| *f*u_Gut_ | 0.06 |  |
| Q_Gut_ | Predicted |  |
| Lag time (h) /CV (%) | 0/30 |  |
| PSA (A^2^) | 69.06 |  |
| Distribution (Minimal PBPK Model) |  |  |
| Single Adjusting Compartment |  |  |
| k_in_ (1/h) | 0 |  |
| K_out_ (1/h) | 0 |  |
| Volume [V_sac_] (L/kg) | 1E-05 |  |
| *V*_ss_ (L/kg) | 0.345 |  |
| CV V_ss_ (%) | 30 |  |
| Tissue : Plasma Partition Coefficients |  |  |
| Kp Liver | 1 |  |
| Elimination |  |  |
| *In Vivo* Clearance |  |  |
| CL_po_ (L/h) &CV (%) | 7.4/40 |  |
| CL_R_ (L/h) | 0.147 |  |
| Active Hepatic Scalar (Net) | 2.07 |  |
| Interaction |  |  |
| Competitive Inhibition |  |  |
| CYP2C8 (Ki/*f*u_mic_) | 2.5/0.87 |  |
| CYP2C9 (Ki/*f*u_mic_) | 10/0.95 |  |
| CYP3A4 (Ki/*f*u_mic_) | 0.015/0.97 |  |
| CYP3A5 (Ki/*f*u_mic_) | 0.109/0.96 |  |
| **Itraconazole** | | |
| Physicochemical properties |  |  |
| Molecular weight (g/mol) | 705.6 |  |
| Log *P* | 4.47 |  |
| Compound type | Monoprotic base |  |
| *pK*_a 1_ | 4.28 |  |
| Blood-to-plasma partition ratio | 0.58 |  |
| Fraction unbound in plasma | 0.016 |  |
| Absorption |  |  |
| Absorption model | First-Order |  |
| User Input |  |  |
| fa/CV (%) | 0.59/30 |  |
| ka (1/h)/CV (%) | 0.6/30 |  |
| *f*u_Gut_ | 0.016 |  |
| *Q*_Gut_/CV (%) | 18.32/12 |  |
| Lag time (h) /CV (%) | 0/30 |  |
| MechPeff model | Predicted |  |
| Distribution (Minimal PBPK Model) |  |  |
| Single Adjusting Compartment |  |  |
| k_in_ (1/h) | 0 |  |
| k_in_ (1/h) | 0 |  |
| Volume [Vsac] (L/kg) | 1E-05 |  |
| *V*_ss_ (L/kg) | Method 1 | Predicted |
| Tissue : Plasma Partition Coefficients |  |  |
| Kp Scalar | 0.3 |  |
| Elimination |  |  |
| Enzyme Kinetics | Recombinant |  |
| CYP1A2 (CL_int_) (μL/min/pmol)/*f*u_mic_ | 1/1 |  |
| CYP3A4 (V_max_/K_m_) (pmol/min/pmol of isoform & μM)/ *f*u_mic_ | 0.065/0.0039/1 |  |
| Active Hepatic Scalar (Net) | 1 |  |
| Interaction |  |  |
| Competitive Inhibition |  |  |
| CYP3A4 (Ki/*f*u_mic_) | 0.0013/1 |  |
| **Erythromycin** | | |
| Physicochemical properties |  |  |
| Molecular weight (g/mol) | 733.9 |  |
| Log *P* | 2.5 |  |
| Compound type | Monoprotic base |  |
| *pK*_a 1_ | 8.8 |  |
| Blood-to-plasma partition ratio | 0.854 |  |
| Fraction unbound in plasma | 0.31 |  |
| Absorption |  |  |
| Absorption model | First-Order |  |
| User Input |  |  |
| fa/CV (%) | 1/30 |  |
| ka (1/h)/CV (%) | 3.58/30 |  |
| *f*u_Gut_ | 1 |  |
| Q_Gut_ | Predicted |  |
| Lag time (h)/CV (%) | 1.37/30 |  |
| Caco-2 (10^-6^ cm/s) (pH 7.4: pH 7.4, Passive&Active) | 1.7 |  |
| Reference |  |  |
| Atenolol_P_app_ (10^-6^ cm/s) | 1.91 |  |
| Propranolol_P_app_ (10^-6^ cm/s) | 147 |  |
| Metoprolol_P_app_ (10^-6^ cm/s) | 145 |  |
| Distribution (Minimal PBPK Model) |  |  |
| Single Adjusting Compartment |  |  |
| k_in_ (1/h) | 0 |  |
| k_out_ (1/h) | 0 |  |
| Volume [Vsac] (L/kg) | 1E-05 |  |
| V_ss_ (L/kg)/CV V_ss_ (%) | 0.75/30 |  |
| Tissue : Plasma Partition Coefficients |  |  |
| Kp Liver | 1 |  |
| Elimination |  |  |
| Enzyme Kinetics | Recombinant |  |
| CYP3A4 (V_max_/K_m_) (pmol/min/pmol of isoform & μM)/*f*u_mic_ | 1.45/14.7/1 |  |
| CYP3A5(V_max_/K_m_) (pmol/min/pmol of isoform & μM)/*f*u_mic_ | 1.25/30.7/1 |  |
| Additional clearance (Liver) |  |  |
| HLM (CL_int_) (μL/min/mg protein) | 13 |  |
| CL_R_ (L/h) | 3.13 |  |
| Active Hepatic Scalar (Net) | 1 |  |
| Interaction |  |  |
| Competitive Inhibition |  |  |
| CYP3A4 (Ki/*f*u_mic_) (μM) | 82/0.909 |  |
| CYP3A4 (K_app_/k_inact_/*f*u_mic_) (μM&1/h) | 23.2/2.25/1 |  |
| CYP3A5 (K_app_/k_inact_/*f*u_mic_) (μM&1/h) | 7.14/0.66/0.972 |  |
| **Fluconazole** | | |
| Physicochemical properties |  |  |
| Molecular weight (g/mol) | 306.3 |  |
| Log *P* | 0.2 |  |
| Compound type | Monoprotic Base |  |
| *pK*_a 1_ | 1.76 |  |
| Blood-to-plasma partition ratio | 1 |  |
| Fraction unbound in plasma | 0.89 |  |
| Absorption |  |  |
| Absorption model |  |  |
| Predicted |  |  |
| *f*u_Gut_ | 0.89 |  |
| Q_Gut_ | Predicted |  |
| Lag time (h)/CV(%) | 0/30 |  |
| Caco-2 (10^-6^ cm/s) (pH 6.5: pH 7.4) Passive&Active | 29.8 |  |
| Reference |  |  |
| Cimetidine_P_app_ | 3.06 |  |
| Propranolol_P_app_ | 27.5 |  |
| Distribution (Minimal PBPK Model) |  |  |
| Single Adjusting Compartment |  |  |
| k_in_ (1/h) | 0 |  |
| K_out_ (1/h) | 0 |  |
| Volume [Vsac] (L/kg) | 1E-05 |  |
| *V*_ss_ (L/kg) | 0.76 |  |
| CV V_ss_(%) | 21 |  |
| Tissue : Plasma Partition Coefficients |  |  |
| Kp Liver | 1 |  |
| Elimination |  |  |
| *In Vivo* Clearance |  |  |
| CL_iv_ (L/h)/CV(%) | 1.21/22 |  |
| CL_R_ (L/h) | 0.86 |  |
| Interaction |  |  |
| Competitive Inhibition |  |  |
| CYP2C9 (Ki/*f*u_mic_) (μM) | 20.4/0.89 |  |
| CYP2C19 (Ki/*f*u_mic_) (μM) | 2/1 |  |
| CYP3A4 (Ki/*f*u_mic_) (μM) | 10.7/1 |  |
| CYP3A5(Ki/*f*u_mic_) (μM) | 84.6/1 |  |
| **Fluvoxamine** | | |
| Physicochemical properties |  |  |
| Molecular weight (g/mol) | 318.3 |  |
| Log *P* | 3 |  |
| Compound type | Monoprotic Base |  |
| *pK*_a 1_ | 8.7 |  |
| Blood-to-plasma partition ratio | 1.5 |  |
| Fraction unbound in plasma | 0.14 |  |
| Absorption |  |  |
| Absorption model |  |  |
| User Input |  |  |
| fa/CV (%) | 1/30 |  |
| ka (1/h)/ CV (%) | 0.7/30 |  |
| *f*u_Gut_ | 0.14 |  |
| Q_Gut_ (L/h) | Predicted |  |
| Lag time (h)/CV(%) | 0/30 |  |
| MDCK II (10^-6^ cm/s) | 31.7 |  |
| Reference |  |  |
| Atenolol_P_app_ (10^-6^ cm/s) | 0.26 |  |
| Propranolol_P_app_ (10^-6^ cm/s) | 40.3 |  |
| Verpamil_P_app_ (10^-6^ cm/s) | 41.5 |  |
| Metoprolol_P_app_ (10^-6^ cm/s) | 29.6 |  |
| Distribution (Minimal PBPK Model) |  |  |
| Single Adjusting Compartment |  |  |
| Volume [Vsac] (L/kg) | 6 |  |
| Q (L/kg) | 0.5 |  |
| *V*_ss_ (L/kg) | 21 |  |
| CV V_ss_(%) | 30 |  |
| Tissue : Plasma Partition Coefficients |  |  |
| Kp Liver | 1 |  |
| Elimination |  |  |
| Enzyme Kinetics | Recombinant |  |
| CYP2D6 (V_max_/K_m_) (pmol/min/pmol of isoform & μM)/*f*u_mic_ | 70/38.6/1 |  |
| Additional clearance (Liver) |  |  |
| HLM (CL_int_) (μL/min/mg protein) | 14 |  |
| Active Hepatic Scalar (Net) | 3 |  |
| Interaction |  |  |
| Competitive Inhibition |  |  |
| CYP1A2 (Ki/*f*u_mic_) (μM) | 0.002/1 |  |
| CYP2C9 (Ki/*f*u_mic_) (μM) | 0.126/1 |  |
| CYP2C19 (Ki/*f*u_mic_) (μM) | 0.006/1 |  |
| CYP2D6 (Ki/*f*u_mic_) (μM) | 0.189/1 |  |
| CYP3A4 (Ki/*f*u_mic_) (μM) | 0.789/1 |  |
| CYP3A5 (Ki/*f*u_mic_) (μM) | 5.82/1 |  |
| **Cimetidine** | | |
| Physicochemical properties |  |  |
| Molecular weight (g/mol) | 252.34 |  |
| Log *P* | 0.48 |  |
| Compound type | Monoprotic Base |  |
| *pK*_a 1_ | 6.9 |  |
| Blood-to-plasma partition ratio | 0.97 |  |
| Fraction unbound in plasma | 0.8 |  |
| Absorption |  |  |
| Absorption model |  |  |
| User Input |  |  |
| fa/CV (%) | 0.92/30 |  |
| ka (1/h) /CV(%) | 0.7/18 |  |
| *f*u_Gut_ | 1 |  |
| Qu_Gut_ | Predicted |  |
| Lag time (h)/CV(%) | 0.15/28 |  |
| Permeability_P_eff,man_ (10^-4^ cm/s) | 0.26 |  |
| Distribution (Full PBPK Model) |  |  |
| *V*_ss_ (L/kg) | Method 2 | Predicted |
| Tissue : Plasma Partition Coefficients |  |  |
| Kp Scalar | 1 |  |
| Elimination |  |  |
| Additional clearance (Liver) |  |  |
| HLM (CL_int_) (μL/min/mg protein) | 2.87 |  |
| CL_R_ (L/h) | 31.6 |  |
| Active Hepatic Scalar (Net) | 1 |  |
| Interaction |  |  |
| Competitive Inhibition |  |  |
| CYP2D6 (Ki/*f*u_mic_) (μM) | 3.5/1 |  |
| CYP3A4 (Ki/*f*u_mic_) (μM) | 25/1 |  |
| Transport (Permeability Ltd.Organs) |  |  |
| Use permeability-limited kidney model (Mech KiM) |  |  |
| CL_PD,basal (mL/min/million proximal tubular cells)_ | 2.61E-05 |  |
| CL_PD,apical (mL/min/million proximal tubular cells)_ | 2.61E-05 |  |
| *f*u_Kidney cell_ | 1 |  |
| *f*u_Urine_ | 1 |  |
| Basal |  |  |
| SLC22A2(J_max_/K_m_ & RAF/REF) | 2170/72.6 & 3 |  |
| SLC22A8(J_max_/K_m_ & RAF/REF) | 1232/161.5 & 3 |  |
| Apical |  |  |
| ABCC4 (J_max_/K_m_ & RAF/REF) | 216/18.2 & 3 |  |
| SLC47As (J_max_/K_m_ & RAF/REF) | 135.5/7.7 & 3 |  |
| **Rifampicin** | | |
| Physicochemical properties |  |  |
| Molecular weight (g/mol) | 823 |  |
| Log *P* | 4.01 |  |
| Compound type | Ampholyte |  |
| *pK*_a 1_ | 1.7 |  |
| *pK*_a 2_ | 7.9 |  |
| Blood-to-plasma partition ratio | 0.9 |  |
| Fraction unbound in plasma | 0.116 |  |
| Absorption |  |  |
| Absorption model |  |  |
| Predicted |  |  |
| *f*u_Gut_ | 0.14 |  |
| Q_Gut_/CV(%) | 9.42/30 |  |
| Lag time (h)/CV(%) | 0/30 |  |
| Caco-2 (10^-6^ cm/s) (pH 6.5: pH 7.4, Passive) | 15 |  |
| Reference |  |  |
| Propranolol_P_app_ (10^-6^ cm/s) | 21.15 |  |
| Distribution (Minimal PBPK Model) |  |  |
| Single Adjusting Compartment |  |  |
| Volume [Vsac] (L/kg) | 1E-05 |  |
| k_in_ (1/h) | 0 |  |
| k_out_ (1/h) | 0 |  |
| *V*_ss_ (L/kg) | 0.42 |  |
| CV V_ss_(%) | 47 |  |
| Tissue : Plasma Partition Coefficients |  |  |
| Kp Liver | 1 |  |
| Elimination |  |  |
| *In Vivo* Clearance | Recombinant |  |
| CL_iv_ (L/h) | 8.7 |  |
| CV (%) | 30 |  |
| CL_R_ (L/h) | 1.26 |  |
| Active Hepatic Scalar (Net) | 1 |  |
| Interaction |  |  |
| Competitive Inhibition |  |  |
| CYP2C8 (Ki/*f*u_mic_) (μM) | 24.5/1 |  |
| CYP3A4 (Ki/*f*u_mic_) (μM) | 15/1 |  |
| Induction/Suppression |  |  |
| CYP1A2 (Ind_max_^/CV(%)/ IndC_50_^/CV(%)/*f*u_inc_/γ) (μM) | 2.7/30/0.1/30/1/1 |  |
| CYP2B6 (Ind_max_^/CV(%)/ IndC_50_^/CV(%)/*f*u_inc_/γ) (μM) | 5.04/30/0.07/30/1/1 |  |
| CYP2C8 (Ind_max_^/CV(%)/ IndC_50_^/CV(%)/*f*u_inc_/γ) (μM) | 6.7/30/0.3/30/1/1 |  |
| CYP2C9 (Ind_max_^/CV(%)/ IndC_50_^/CV(%)/*f*u_inc_/γ) (μM) | 6/30/0.1/30/1/1 |  |
| CYP2C19(Ind_max_^/CV(%)/ IndC_50_^/CV(%)/*f*u_inc_/γ) (μM) | 5.5/30/0.32/30/1/1 |  |
| CYP3A4 (Ind_max_^/CV(%)/ IndC_50_^/CV(%)/*f*u_inc_/γ) (μM) | 16/30/0.32/30/1/1 |  |
| CYP3A5 (Ind_max_^/CV(%)/ IndC_50_^/CV(%)/*f*u_inc_/γ) (μM) | 16/30/0.32/30/1/1 |  |
| **Efavirenz** | | |
| Physicochemical properties |  |  |
| Molecular weight (g/mol) | 315.68 |  |
| Log *P* | 4.02 |  |
| Compound type | Monoprotic Acid |  |
| *pK*_a 1_ | 10.2 |  |
| Blood-to-plasma partition ratio | 0.74 |  |
| Fraction unbound in plasma | 0.029 |  |
| Absorption |  |  |
| Absorption model |  |  |
| User Input |  |  |
| fa/CV (%) | 0.67/15 |  |
| ka (1/h)/CV (%) | 0.41/15 |  |
| *f*u_Gut_ | 0.005 |  |
| Q_Gut_ |  | Predicted |
| Lag time (h)/CV(%) | 0.36/30 |  |
| PSA (A^2^) | 38.33 |  |
| Distribution (Minimal PBPK Model) |  |  |
| Single Adjusting Compartment |  |  |
| Volume [Vsac] (L/kg) | 1.1 |  |
| k_in_ (1/h) | 0.29 |  |
| k_out_ (1/h) | 0.9 |  |
| *V*_ss_ (L/kg) | Method 2 | Predicted |
| Tissue : Plasma Partition Coefficients |  |  |
| Kp Scalar | 0.155 |  |
| Elimination |  |  |
| Enzyme Kinetics | Recombinant |  |
| CYP1A2 CL_int_ (μL/min/pmol of isoform)/*f*u_mic_ | 0.03/1 |  |
| CYP2B6 CL_int_ (μL/min/pmol of isoform)/*f*u_mic_ | 1.36/1 |  |
| CYP2A6 CL_int_ (μL/min/pmol of isoform)/*f*u_mic_ | 0.47/1 |  |
| CYP3A4 CL_int_ (μL/min/pmol of isoform)/*f*u_mic_ | 0.01/1 |  |
| Additional clearance (Liver) |  |  |
| HLM (CL_int_) (μL/min/mg protein) | 0.694 |  |
| Active Hepatic Scalar (Net) | 1 |  |
| Interaction |  |  |
| Induction/Suppression |  |  |
| CYP2B6 (Ind_max_^/CV(%)/ IndC_50_^/CV(%)/*f*u_inc_/γ) (μM) | 6.2/30/1.2/30/0.15/1 |  |
| CYP3A4 (Ind_max_^/CV(%)/ IndC_50_^/CV(%)/*f*u_inc_/γ) (μM) | 9.9/30/3.8/30/0.15/1 |  |

**Table S3** Summary of the PB-201 PBPK model parameters

| **Parameter** | **Input Value** | **Source** |
| --- | --- | --- |
| Physicochemical properties |  |  |
| Molecular weight (g/mol) | 432.44 |  |
| Log *P* | 1.38 | Measured |
| Compound type | Monoprotic base |  |
| *pK*_a_ | 10.41 | Measured |
| Blood-to-plasma partition ratio | 0.77 | Measured |
| Fraction unbound in plasma | 0.303 | Measured |
| Absorption |  |  |
| Absorption model | ADAM |  |
| Permeability Assay | Caco-2 |  |
| Apical pH : Basolateral pH | 7.4 : 7.4 |  |
| Activity |  |  |
| Caco-2(10^-6^cm/s) (PB-201) | 8.88 | Measured |
| Caco-2(10^-6^cm/s) (Propranolol) | 29.4 | Measured |
| DLM Model Options | Particle Population Balance Model |  |
| Formulation | Immediate release |  |
| Solid State Specific Parameters | Solid state 1 |  |
| Dissolution Type | Solubility |  |
| Solubility Type | Intrinsic solubility |  |
| Solubility (mg/mL) | 0.000123 | Fitted |
| Salt Limited Solubility Model | Solubility Factors |  |
| Solubility Factors | 1000 | Default |
| Supersaturation Precipitation Model | First order | Default |
| Particle size distribution |  |  |
| Monodispersed Radius (μm) | 10.0 | Default |
| Particle density (g/mL) | 1.2 | Default |
| Particle population balance model options (Simulation Parameters) |  |  |
| Number of Particle Size Bins (Simulation) | 30 |  |
| Radius Bounds (Simulation) (μm) |  |  |
| Minimum | 0.1 |  |
| Maximum | 11 |  |
| h_eff_ method selected | Hintz-Johnson |  |
| h_eff_ cut-off type | Default |  |
| DLM Scalar | All segments |  |
| DLM Scalar values | 1.0 | Default |
| Log *K* _m:w, neutral_ | 3.3112 | Predicted |
| Log *K* _m:w, ion_ | 2.3112 | Predicted |
| Distribution |  |  |
| Distribution model | Full PBPK model |  |
| *V*_ss_ (L/kg) | 1.884 | Predicted |
| Prediction Method | Method 2 |  |
| Tissue : Plasma Partition Coefficients |  | Predicted |
| *K*_p_ scalar | 1.5 | Fitted |
| Elimination |  |  |
| Clearance type | Enzyme kinetics |  |
| Intrinsic clearance of CYP 1A2 (μL/min/pmol) | 0.068 | Measured |
| *ISEF* of CYP 1A2 | 0.336 | Fitted |
| Intrinsic clearance of CYP 2B6 (μL/min/pmol) | 0.072 | Measured |
| *ISEF* of CYP 2B6 | 1.452 | Fitted |
| Intrinsic clearance of CYP 2C8 (μL/min/pmol) | 0.06 | Measured |
| *ISEF* of CYP 2C8 | 1.452 | Fitted |
| Intrinsic clearance of CYP 2C9 (μL/min/pmol) | 0.144 | Measured |
| *ISEF* of CYP 2C9 | 0.548 | Fitted |
| Intrinsic clearance of CYP 2C19 (μL/min/pmol) | 0.113 | Measured |
| *ISEF* of CYP 2C19 | 1.452 | Fitted |
| Intrinsic clearance of CYP 2D6 (μL/min/pmol) | 0.145 | Measured |
| *ISEF* of CYP 2D6 | 0.66 | Fitted |
| Intrinsic clearance of CYP 3A4 (μL/min/pmol) | 0.141 | Measured |
| *ISEF* of CYP 3A4 | 0.33 | Fitted |
| Typical renal clearance (L/h) | 0.099 | Measured |
| Additional Hep CL_int_ (μL/min/10^6^) | 2.50 | Fitted |

**Figures:**


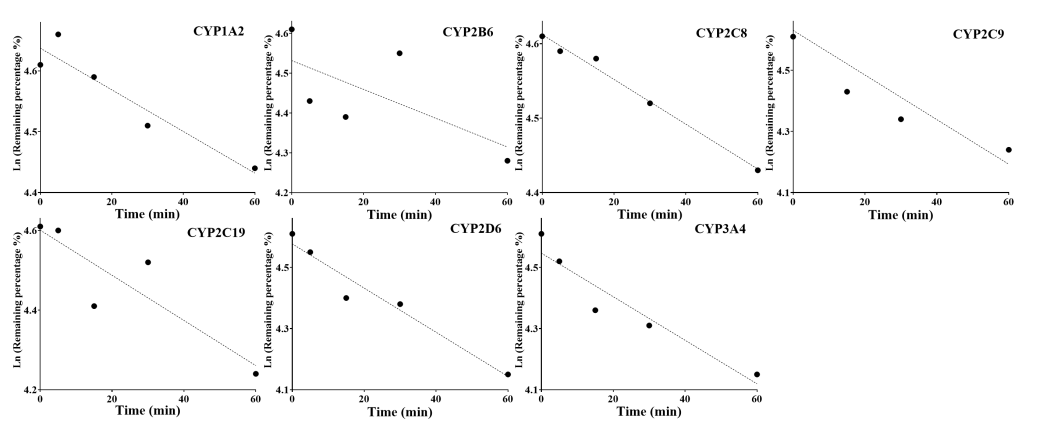


**Figure S1** Remaining percentage of PB-201 in human recombinant CYP isoforms incubation systems.


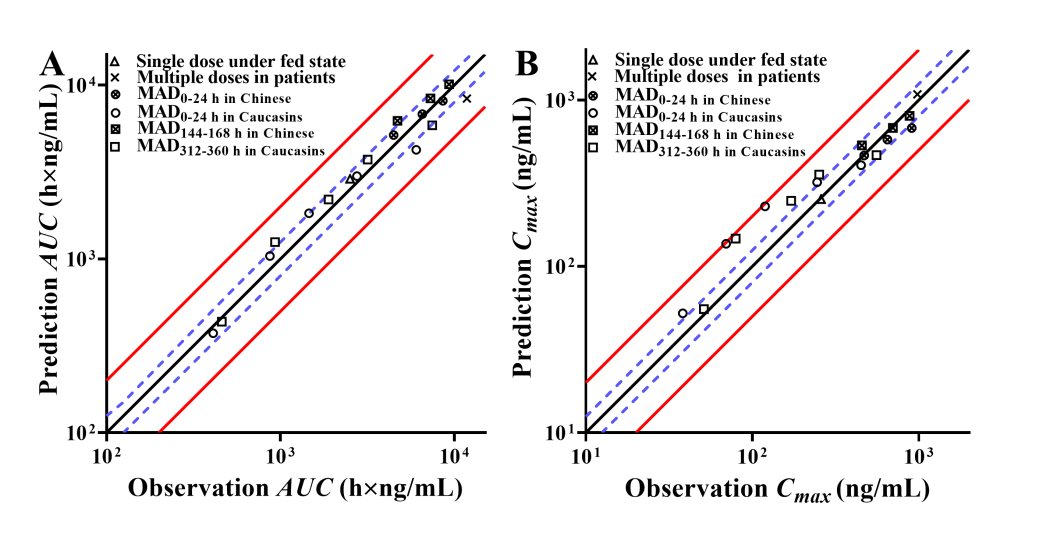


**Figure S2** Validated results of pharmacokinetic parameters according to the single dose under fed state, multiple ascending doses (MAD) in Chinese and Caucasians, and multiple doses in T2MD patients (A: *AUC*; B: *C*_max_; the red solid lines are predefined 2-fold boundaries; the blue dashed lines are predefined 1.25-fold boundaries; and the black solid line represents the boundary where prediction results are equal to the observation ones).

1. QD: Quaque Die; BID: Bis In Die; QID: Qualer In Die [↑](#footnote-ref-1)
